# Intensity modulation of frequency-specific optoacoustic stimulation at the peripheral hearing level

**DOI:** 10.1016/j.zemedi.2025.12.004

**Published:** 2025-12-30

**Authors:** Patricia Dries, Hubert H. Lim, Marius Hinsberger, Katharina Sorg, Lukas Pillong, Achim Langenbucher, Bernhard Schick, Gentiana I. Wenzel

**Affiliations:** a Saarland University, Faculty of Medicine, Department of Otolaryngology, Kirrbergerstr. 100, 66421 Homburg, Germany; b University of Minnesota, Department of Biomedical Engineering, United States; c Saarland University, Faculty of Medicine, Experimental Ophthalmology, Germany

**Keywords:** Auditory prosthesis, laser, optoacoustic, audiology, otolaryngology

## Abstract

Laser light applied to the tympanic membrane (TM) induces mechanical vibrations that activate the auditory system. For controlled optoacoustic stimulation, a quantitative characterization of the mechanical properties of the TM and their relation to stimulation intensity is required. We therefore investigated which optical stimulation parameters induce vibratory amplitudes comparable to those caused by acoustic stimulation.

TM vibration velocity was recorded *ex vivo* using a scanning laser Doppler vibrometer. Mechanical parameters of the TM, including effective compliance and exponential decay constants (τ), were determined from single-pulse laser stimulation. In addition, vibration amplitudes were quantified in response to sinusoidally amplitude-modulated laser pulse trains at different laser modulation rates (LMR). Laser modulation rates of 1, 2, 4, 8 and 10 kHz, and laser pulse repetition rates (LPR) of 32 kHz and 50 kHz were applied. *Ex vivo* measurements were compared with frequency-specific *in vivo* recordings from the central nucleus of the inferior colliculus (ICC).

The extracted mechanical parameters, including compliance and τ, were consistent across stimulation conditions. *Ex vivo* measurements showed that the maximal displacement amplitude at the umbo decreased with increasing LMR and approached values comparable to those obtained with pure-tone acoustic stimulation up to 60 dB SPL. These intensity-dependent mechanical responses were reflected by corresponding neural activity at the ICC level *in vivo*, demonstrating consistent optoacoustic coupling across mechanical and physiological domains.

## Introduction

The auditory system senses mechanical stimuli with multiple frequencies from the environment. These signals consist of sound pressure waves transmitted through the outer ear canal to the vibratory structures of the middle ear and, ultimately, to the inner ear (the cochlea). The cochlea transduces sound information into electrical impulses and transmits them to the central nervous system.

Worldwide, an estimated 430 million people suffer from disabling hearing impairment with at least moderate hearing loss in the better hearing ear, including 34 million children [1]. This number is rising due to a growing global population, longer life expectancy, and increasing noise exposure from environmental, workplace, and recreational activities. Unaddressed hearing loss poses an annual global cost of 750 billion international dollars. Interventions to prevent, identify, and compensate for or cure hearing loss are cost-effective and can provide substantial benefit to individuals [1].

If hearing impairment cannot be treated by middle ear surgery, current therapy for hearing loss involves delivering mechanical energy via a conventional hearing aid or electrical energy via a cochlear implant to the ear.

Despite technological advances that have enhanced the performance of traditional auditory prostheses, many people with hearing impairments still do not receive adequate compensation for their hearing loss with these devices.

Current hearing devices based on mechanical, magnetic, or electrical energy present several limitations, such as acoustic feedback, user discomfort due to the occlusion effect, recurrent inflammation of the external auditory canal, and inadequate frequency resolution [2].

This has prompted the search for alternative approaches that more closely mimic the natural function of the auditory system and yield improved outcomes for individuals with hearing deficits, leading to the introduction of a new form of energy: laser light.

This innovative approach offers the potential for targeted activation of auditory structures without many of the drawbacks associated with conventional devices.

The advantage of laser light is its ability to be focused with high spatial precision to specific structures. The first non-ablative laser application to the inner ear, the cochlea, was reported in 2004 [3] demonstrating that laser irradiation of the cochlea can alter collagen organization within the basilar membrane. This method may be used to modulate cochlear mechanics and alter cochlear tuning. Other current approaches using light stimulation for the peripheral auditory system target patients with severe to profound sensorineural hearing loss (i.e., significant loss of functional hair cells) [4], [5], [6], [7], [8], [9], [10], [12].

However, a large proportion of hearing-impaired individuals have conductive, sensorineural, or combined hearing loss with residual hearing that can still be activated. To develop a useful auditory prosthesis for this patient group, controlled modulation of the incoming optical signal is mandatory to activate the peripheral hearing organ precisely.

In previous work, we demonstrated optoacoustic, frequency-specific activation of the peripheral auditory system by applying laser pulse trains with varying amplitudes to the tympanic membrane (TM) as well as to different locations within the middle and outer ear [11], [13]. Using a pulse-amplitude modulation approach, we demonstrated *in vitro* that this optical stimulation reliably induces vibrations of the peripheral auditory organ, as quantified by laser Doppler vibrometer recordings. The measured responses were validated using a convolution-based model that employed single-pulse laser Doppler vibrometer recordings as the system’s impulse response. Furthermore, we translated this stimulation strategy to *in vivo* experiments and recorded electrophysiological responses at the midbrain level, revealing a clear frequency shift associated with different laser modulation rates [13].

These promising results could be an important step towards the development of a new type of laser-based hearing device for complex acoustic sounds such as speech and music, which consist of multiple frequencies.

The next important dimension of sound is the intensity. The output of the optical hearing device must lead to impressions that differ between soft sounds like a whisper and louder ones like the ocean in a wild storm.

Work by Maier et al. in human cadaveric temporal bones demonstrated that optoacoustic stimulation at the round window can induce pressure levels comparable to 140 dB eq SPL at low frequencies (<200 Hz) and up to 90 dB eq SPL at 1 kHz [14].

While our previous studies demonstrated frequency-specific optoacoustic activation of the peripheral auditory system and corresponding neural responses in the midbrain, the mechanical pathway linking absorbed laser energy to TM motion has not yet been quantitatively characterized. In particular, the relationship between laser-induced temperature transients and the resulting mechanical deformation of the tympanic membrane remains insufficiently described.

To address this issue, we relate a simplified thermoelastic interpretation to experimentally derived mechanical parameters of the TM, including compliance and relaxation time constants. This allows a first-order, physically guided estimation of how absorbed optical energy translates into mechanical displacement across different laser intensities. By complementing our *in vitro* LDV measurements with *in vivo* recordings, this approach supports a more coherent physical interpretation of the observed frequency specificity and intensity scaling in optoacoustic stimulation.

Building on these findings, the present study extends our previous work [13] by analyzing harmonic and pulse-rate related components of the optoacoustic response across different laser intensities and by determining equivalent acoustic SPL levels. By relating the observed mechanical responses of the TM to corresponding neural activity, this study provides a clearer physical interpretation of intensity-dependent optoacoustic stimulation at the tympanic membrane.

In total, we investigated whether optical stimulation at the TM level can induce vibratory amplitudes comparable to those evoked by acoustic stimulation across a broad range of intensities, and to interpret these responses within the simplified thermoelastic framework linking laser energy deposition to mechanical displacement.

## Material and methods

### Summary of the experimental setup

The experimental setup has been previously described in [13] by the same authors. To avoid the need for additional animal experiments, the raw *in vitro* and *in vivo* recordings originate from the same experimental sessions as described in [13], but all modelling and analyses presented here were performed *de novo* with updated processing steps. No previously published derived data or figures were reused unless explicitly cited.

Albino guinea pigs (Charles River Laboratories, Sulzfeld, Germany) were selected following approval from the Animal Welfare Office and Central Veterinary Office of Saarland University (TV27/2011; TV41/2015). Data is reported from 22 animals: 9 for *in vitro* and 13 for *in vivo* experiments. For thermoelastic characterization, data from 3 explanted guinea pig temporal bones were obtained *in vitro*.

Green light (532 nm) from a Neodymium-doped Yttrium Orthovanadate (Nd: YVO_4_) laser system (Model “INCA”, Xiton Photonics GmbH, Kaiserslautern, Germany) was delivered through a 365 µm multimode fiber onto the umbo, the central attachment point of the TM in the guinea pig. Two stimulation paradigms were used.

(1) Single-pulse stimulation for thermoelastic characterization.

To quantify the mechanical response of the TM to localized thermoelastic expansion, isolated nanosecond pulses were applied with energies between 1 µJ and 40 µJ, in a logarithmic scale with 5 steps to cover a broad dynamic range. These single-pulse measurements served as the basis for deriving mechanical compliance and relaxation time constants by probing the membrane’s response to an impulsive optoacoustic drive.

(2) Sinusoidally modulated optoacoustic stimulation for frequency-specific response analysis.

For assessing frequency-dependent mechanical and neural activation, the laser was operated in pulsed mode at laser-pulse rates (LPR) of 32 kHz and 50 kHz. The pulse train was sinusoidally amplitude-modulated at defined laser-modulation rates (LMR) of 1, 2, 4, 8, and 10 kHz. This LPR/LMR configuration produces a quasi-continuous acoustic drive whose envelope oscillates sinusoidally, thereby enabling frequency-specific probing of the middle ear and auditory pathway while maintaining the thermoelastic generation mechanism of each individual pulse.

Depending on the experimental condition, laser output levels at the fiber tip ranged on a logarithmic power scale between 30 and 320 mW.

**Fig. 1:**
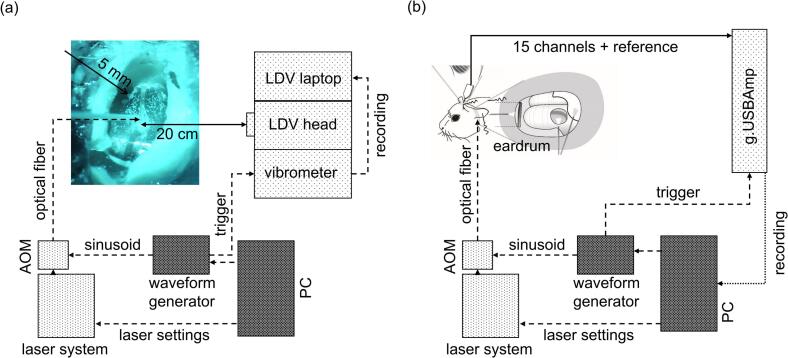
Experimental setup (a) Umbo laser Doppler vibrometer setup in excised species (in vitro) (b) Experimental setup in living animals in vivo. Source (adapted): Based on Stahn P., et al. Frequency-specific activation of the peripheral auditory system using optoacoustic laser stimulation. Scientific Reports (2019) [13]. Licensed under Creative Commons Attribution 4.0 (CC BY 4.0). Adapted by the first author.

Mechanical responses of the TM were recorded using a scanning laser Doppler vibrometer (LDV, Polytec GmbH, Waldbronn, Germany). The LDV beam was aligned to the umbo as central part of the TM and measured single-point velocity time traces concurrent with optical stimulation. LDV signals were acquired with a sampling rate sufficient to resolve the applied modulation frequencies and pulse structure; recorded traces were baseline-corrected, band-pass filtered around the modulation frequency where appropriate, and averaged across repeated trials to improve signal-to-noise ratio (SNR). Temporal synchronization between the laser drive, LDV acquisition, and electrophysiological recording was achieved via a common trigger signal, enabling direct stimulus-response correlation. At the beginning of the LDV-recordings in each experiment, we applied pure tones (1 kHz, 2 kHz, 4 kHz, 8 kHz, and 10 kHz) with 0 dB SPL as well as 60 dB SPL, 70 dB SPL, and 80 dB SPL. This data allowed us to compare the optical-induced vibrations of the TM with the acoustic-induced TM vibrations as well (Fig. 1a).

To assess central neural activation, electrophysiological recordings were performed at the level of the inferior colliculus (ICC). Multi-unit activity was recorded using a multichannel A1x16 electrode (NeuroNexus Technologies, Ann Arbor, MI, USA) inserted into ICC laminae corresponding to low- to mid-frequency regions. Neural responses were quantified as stimulus-triggered changes in firing rate relative to baseline, enabling direct comparison between peripheral mechanical responses measured by laser Doppler vibrometry and central auditory activation at the ICC level (Fig. 1b).

The optical and recording hardware were not modified for this study. Rather, we extended the analysis of the stimulus–response relationship across different modulation frequencies and intensity levels. Full technical specifications of the laser system, LDV configuration, calibration procedures, optical path, data acquisition hardware, and ICC recording methodology are provided in [13].

### Fundamentals of optoacoustic stimulation

Single-pulse optoacoustic stimulation was used to characterize the mechanical response of the TM under nanosecond laser excitation. These measurements yielded two important biomechanical parameters: mechanical compliance, describing displacement per unit absorbed energy, and the mechanical relaxation time τ, describing the decay of the dominant post-stimulus vibration. These metrics provide a compact link between optical energy deposition, transient TM motion, and the responses evoked during subsequent modulated stimulation experiments.

#### Thermoelastic excitation of the tympanic membrane

Nanosecond pulses generate thermoelastic expansion in the superficial TM layers under conditions of stress and thermal confinement. The induced pressure field $p\left(r,t\right)$ follows

$$\nabla^{2}p-\frac{1}{c^{2}}\frac{\partial^{2}p}{\partial t^{2}}=-\frac{\beta }{C_{p}}\frac{\partial H}{\partial t},$$

where c refers to the sound speed, β to the thermal expansion coefficient, $C_{p}$ to the heat capacity, and $H\left(r,t\right)$ to the heating rate.

For short excitation (ns laser pulses), ∂H/∂t becomes impulse-like, yielding the initial pressure rise

$$p_{0}=\Gamma A_{e},$$

with Γ the Grüneisen parameter and $A_{e}$ the absorbed energy density.

This pressure impulse induces a rapid outward displacement followed by damped mechanical oscillation.

#### Temporal components of the measured response

LDV recordings exhibited three reproducible features:

(1) a slow, non-oscillatory thermal deformation over several milliseconds
(2) a dominant kHz-range oscillatory mode with exponential amplitude decay
(3) smaller secondary modal contributions.

To avoid over-parameterization of individual trials, the analysis focuses on two robust descriptors: maximal peak displacement relative to the pulse energy (compliance) and the relaxation constant τ of the dominant oscillatory mode.

### Quantification of compliance and mechanical relaxation time τ

#### Signal preprocessing

Each trace was cropped to the post-stimulus interval. Slow thermal drift was removed by subtracting a linear trend line fitted to a late (>2.5 - 3 ms) segment in terms of minimizing the root-mean-squared fit error. A mild zero-phase high-pass filter (20 - 40 Hz, 2-pole Butterworth) suppressed residual baseline fluctuations while preserving the oscillatory mode.

#### Mechanical compliance

Compliance was defined as

$$C_{m}=\frac{max|x(t)|}{E_{\text{pulse}}},$$

reflecting deformation per unit optoacoustic driving force. Values were computed separately for each energy level and animal.

#### Envelope extraction and decay segmentation

The amplitude envelope of the de-trended and high-pass-filtered trace was obtained as the smoothed magnitude signal

$$E\left(t\right)=\text{sgolayfilt}(|x_{\text{hp}}\left(t\right)|,3,w),$$

where sgolayfit() refers to the Savitzky-Golay sliding window filter, avoiding Hilbert-based distortions in noisy or multi-modal recordings, and de-trended denotes removal of slow baseline drift via low-order polynomial subtraction to isolate the oscillatory component.

The decay interval is initialized at the maximum envelope, a candidate range between 95 % and 20 % of the peak was automatically screened. Because the dominant umbo mode decays within approximately 0.2 - 0.4 ms, a minimum interval of 0.4 - 0.6 ms was required to ensure that at least one full time constant was captured while avoiding noise-floor contamination at later time points.

#### Estimation of τ

The logarithmized envelope was fitted by

$$ln\left(E\left(t\right)\right)=at+b,\tau =-\frac{1}{a}.$$

**Fig. 2:**
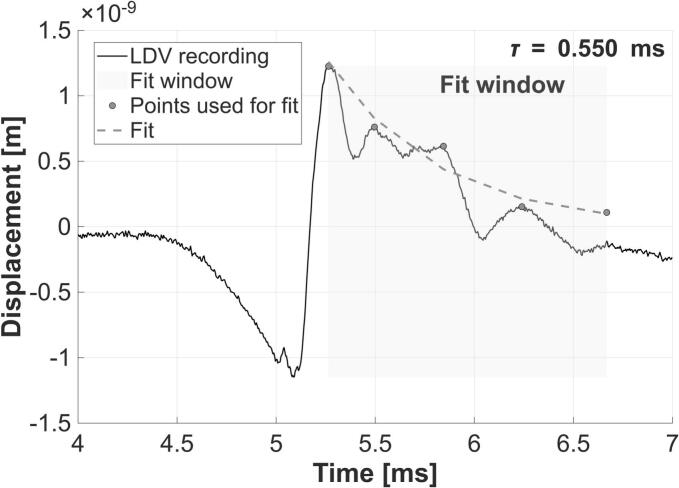
Laser-evoked tympanic membrane displacement and extraction of mechanical relaxation constant τ. Representative LDV displacement waveform following a single 10-ns laser pulse delivered to the umbo. The black trace shows the band-pass–filtered mechanical response, comprising an initial thermoelastic expansion followed by a damped oscillatory motion of the tympanic membrane. The point markers indicate the detected peaks used for estimating the decay of the dominant vibration mode. The shaded region denotes the automatically selected fitting interval, defined as the time interval with strictly decaying signal envelope. The dashed line shows the exponential fit to the peak amplitudes, from which the mechanical relaxation constant τ is derived.

In the linearized fit $ln(E\left(t\right))=at+b$, the slope a represents the decay rate of the oscillatory envelope and is directly related to the mechanical relaxation time τ=-1/a. The intercept $b=ln(E_{0}$) corresponds to the initial envelope amplitude at the beginning of the fit window and does not affect the estimation of τ (Fig. 2).

Fits with non-negative slope or $R^{2}<0.80\;\;$ were rejected. This conservative procedure ensures that τ reflects genuine single-mode mechanical relaxation.

#### Interpretation

The initial displacement amplitude scales with the optoacoustically induced pressure $p_{0}\propto A_{e}$, making $C_{m}$ a measure of TM sensitivity to optical forcing.

The relaxation time τ characterizes the dissipation of mechanical energy through viscoelastic losses, acoustic radiation, and modal coupling. Together, compliance and τ describe the amplitude and temporal dynamics of the TM’s response to laser excitation.

### *In vitro* and *in vivo* data analysis of frequency-specific laser pulse stimulation

The velocity signals acquired with the LDV were band-pass filtered between 300 and 120,000 Hz to retain both the modulation envelope and pulse-related spectral components and averaged across runs to improve signal robustness. Displacement was subsequently derived by numerical integration of the velocity traces. For each stimulus condition, the resulting displacement signals were transformed into the frequency domain using a fast Fourier transform (FFT) to quantify frequency-specific mechanical responses.

**Fig. 3:**
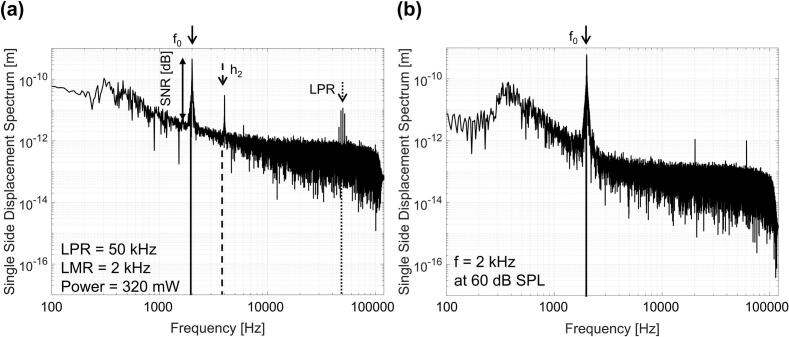
Single side displacement spectrum (a) The displacement spectrum of the umbo vibration after optical stimulation with 2 kHz LMR/32 kHz LPR and 320 mW demonstrated a peak at the LMR f0, and additional peaks at the second harmonic (h2) and at the LPR. (b) The displacement spectrum of the umbo vibration after acoustic stimulation with a 2 kHz sinusoid at 60 dB SPL. Source (adapted): Based on Stahn P., et al. Frequency-specific activation of the peripheral auditory system using optoacoustic laser stimulation. Scientific Reports (2019) [13]. Licensed under Creative Commons Attribution 4.0 (CC BY 4.0). Adapted by the first author.

For optoacoustic stimulation, the fundamental frequency (f_0_) was defined as the laser modulation rate (LMR) of the amplitude-modulated pulse train and represents the main mechanically encoded stimulus frequency. In addition to f_0_, higher-order spectral components were evaluated, including the second harmonic (2f_0_) and components at the laser pulse repetition rate (LPR) (Fig. 3).

For acoustic stimulation, the fundamental frequency f_0_ corresponded to the frequency of the applied sinusoidal sound stimulus. Spectral amplitudes at f_0_ and its harmonics were extracted from the displacement spectra and used for quantitative comparison between optoacoustic and acoustic stimulation conditions.

To estimate the noise level, we calculated the average spectral magnitude within a narrow window adjacent to LMR but excluding the stimulus line (typically ± 50 - 100 Hz, depending on frequency resolution).

The SNR was then computed as:

$$\text{SNR}=20{log}_{10}\left(\frac{A_{\text{LMR}}}{A_{\text{noise}}}\right)$$

where $A_{\text{LMR}\;}$ is the amplitude at the modulation frequency and $A_{\text{noise}}$ the mean noise-floor amplitude in the surrounding band. The same process was applied to the additional peaks at the second harmonic as well as at the LPR.

Neural recordings from the inferior colliculus (ICC) were processed offline in MATLAB® (R2025a, The MathWorks Inc., Natick, USA). Raw signals were high-pass filtered, and neural activity was extracted using a threshold-based spike detection algorithm. For each stimulus, the driven spike rate (DSR) was calculated by subtracting spontaneous activity measured in a pre-stimulus window from the total spike count within the post-stimulus interval.

Electrode placement was verified using acoustically evoked frequency response maps (FRMs) obtained with pure tones at multiple intensity levels. The best frequency (BF) for each channel was determined from the frequency–level combination that elicited the maximal response.

**Fig. 4:**
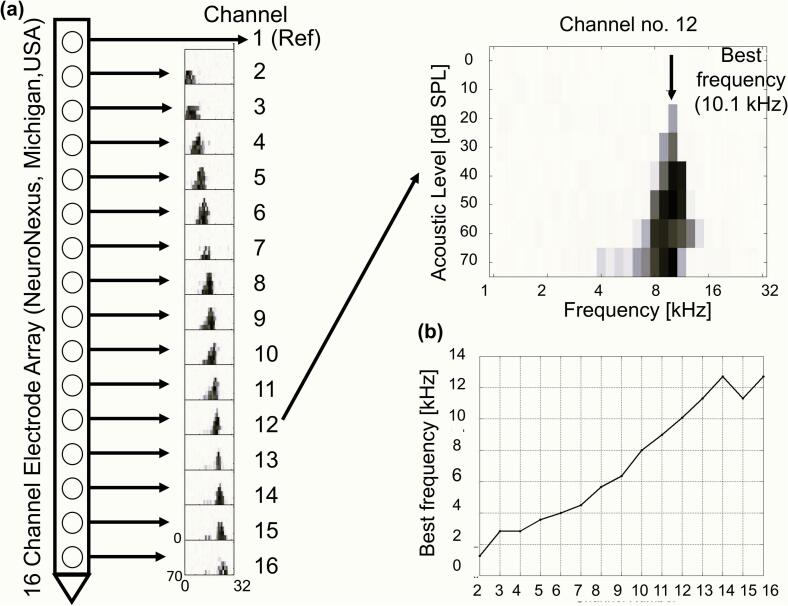
Recordings from the central nucleus of the inferior colliculus (a) Frequency response map compared to the 16-channel electrode leading to the best frequency per channel mapping (b) Acoustic best frequency vs. channel mapping before optical stimulation. Source (adapted): Based on Stahn P., et al. Frequency-specific activation of the peripheral auditory system using optoacoustic laser stimulation. Scientific Reports (2019) [13]. Licensed under Creative Commons Attribution 4.0 (CC BY 4.0). Adapted by the first author

For optical stimulation, the same electrode positions and processing pipeline were used. Responses were quantified as DSR across different laser power levels and modulation frequencies. These values were normalized to the maximum response per channel to construct optical tuning curves (OpTCs). Comparison across electrode depths allowed identification of lamina-specific activation patterns (Fig. 4).

This approach enables a direct comparison between acoustically and optically evoked ICC responses while maintaining a consistent recording and analysis framework.

## Results

### Determination of mechanical parameters after laser pulse stimulation of the tympanic membrane

Laser-induced single-pulse stimulation of the TM produced measurable surface displacements for all tested pulse energies between 6.3 µJ and 40 µJ in 5 steps on a logarithmic scale.

Mechanical compliance could be quantified for all stimulus energies and all animals because the peak displacement preceding modal interference was present in every recording. In contrast, valid relaxation time constants (τ) were only obtained when the dominant oscillatory component exhibited a clear, monotonically damped envelope. Recordings with irregular decay, caused by multi-modal superposition, secondary rebounds, or insufficient SNR, did not meet the predefined inclusion criteria and were excluded from τ estimation. This selective retention of τ values is expected for lightly damped biological structures with spatially distributed modes and guarantees that the reported τ values reflect genuine single-mode decay rather than composite or drift-dominated signals.

Importantly, τ remained highly consistent across energies. This energy-invariance supports the interpretation of τ as an intrinsic mechanical damping parameter of the TM–umbo system rather than an artifact of pulse amplitude or thermal load.

#### Energy–displacement relationship and mechanical compliance

Mechanical compliance was successfully extracted for all recordings across animals and stimulus energies. Compliance values showed moderate intra- and inter-animal variability but remained within a single order of magnitude, consistent with the expected range of nanometer-scale umbo displacements under optoacoustic stimulation.

For Animal 1, compliance values ranged from 0.37·10^–4^ to 2.27·10^–4^ m/J, yielding a mean of 0.86·10^–4^ ± 0.75·10^–4^ m/J (SD; N=5).

For Animal 2, the values ranged from 0.75·10^–4^ m/J to 1.74·10^–4^ m/J, with a mean of 1.33·10^–4^ ± 0.403·10^–4^ m/J (SD; N=5).

Animal 3 exhibited the widest spread (0.47·10^–4^ m/J to 3.34·10^–4^ m/J), with a mean of 1.52·10^–4^ ± 1.06·10^–4^ m/J (SD; N=5), likely reflecting anatomical variations or minor differences in LDV alignment.

Across all animals, compliance values clustered between 0.75·10^–4^ m/J and 1.52·10^–4^ m/J, with inter-animal variations below a factor of two. This level of variation is consistent with reported respective geometric and mechanical benchmarks of the guinea-pig TM and supports the suitability of compliance as an energy-independent descriptor of TM sensitivity.

#### Mechanical relaxation time τ

Across all animals, valid relaxation time constants τ were obtained only for recordings in which the dominant post-stimulus oscillation exhibited a clean, monotonically decaying envelope. Recordings dominated by thermal drift, multimodal interference, or insufficient SNR did not satisfy the predefined criteria and were therefore excluded from τ estimation. The retained τ values thus represent high-confidence measurements of the intrinsic damping behavior of the TM–umbo system.

Across animals, valid τ estimates exhibited a characteristic range but remained highly consistent within each individual. In the first animal, averaging the three valid decay intervals resulted in a relaxation time constant of τ = 0.297 ± 0.022 ms (SD; N=3), reflecting a narrowly clustered set of values and indicating a well-defined, lightly damped oscillatory mode. In the second animal, the three available measurements yielded τ = 0.430 ± 0.116 ms (SD; N = 3). The larger variation here suggests stronger modal superposition or subtle differences in local viscoelastic loading compared with the first animal. For the third animal, a single low-energy estimate was excluded due to clear drift contamination; the two remaining, highly consistent decay intervals resulted in τ = 0.210 ± 0.003 ms (SD; N = 2), matching the lower end of the τ distribution observed in the other animals.

When considering all valid measurements, relaxation times clustered within a compact range of 0.20–0.43 ms, reflecting a common mechanical damping regime across animals. These values are consistent with the lightly damped low-kHz umbo resonance described in classical LDV and middle-ear impedance studies of the guinea pig.

Inter-animal differences, on the order of a few hundred microseconds, likely reflect normal biological variation in TM geometry, tension, and ossicular constraints rather than fundamental biomechanical differences.

### LDV *in vitro* measurements

#### Signal-to-noise ratio at the peak for different LMR and LPR combinations

We observed a linear transfer function across laser modulation rates from 1 kHz to 10 kHz. The SNR depended strongly on the applied laser power.

**Fig. 5:**
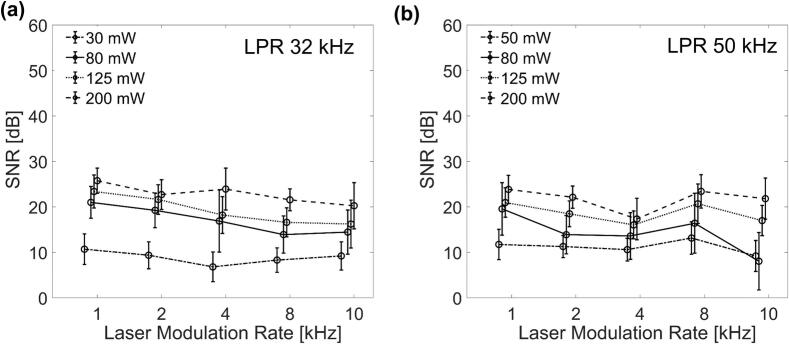
Signal-to-noise ratio (SNR) transfer function in vitro For the LPR of 32 kHz, the SNR tends to decrease with increasing LMR (a). For the LPR of 50 kHz, the SNR tends to decrease for middle LMR (4 kHz), the SNR at lower (1 kHz and 2 kHz) and higher (8 kHz and 10 kHz) is similar (b).

At the lowest stimulation level (30 mW for 32 kHz), SNRs ranged from 6.80 dB SNR at 4 kHz LMR to 10.69 dB SNR at 1 kHz LMR. In contrast, at the highest stimulation level (200 mW at 32 kHz), SNRs ranged from 20.25 dB to 25.75 dB, showing both higher overall response quality and less variability (Fig. 5).

This indicates that both the strength and consistency of the mechanical response increase with laser power, while the modulation rate shows minor impact across the tested range.

#### Growth function of the main frequency peak and the distortion peaks

**Fig. 6:**
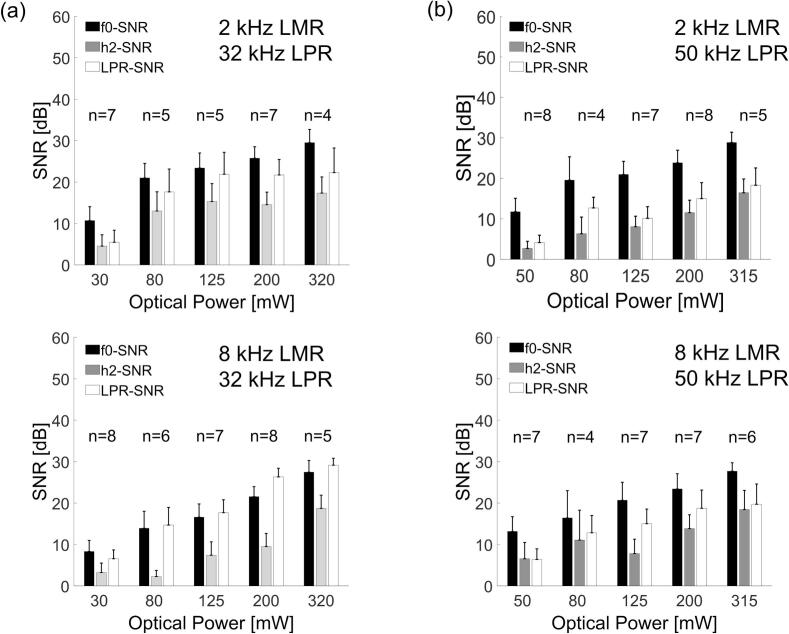
Growth functions in vitro The black bars in the graph indicate the signal-to-noise ratio (SNR) of the main frequency (f0), while the grey and white bars show disturbances at the second harmonic and at the laser pulse rate (LPR) frequency (a) 32 kHz and (b) 50 kHz, respectively. All three signals increase proportionally: as the SNR of the main frequency increases, disturbances at the second harmonic and LPR also increase.

The SNR of the main frequency peak ranges from 8.31 dB to 29.5 dB, while the additional peaks range from 2.69 to 18.72 dB for the second harmonic and to 29.15 dB for the LPR distortion peak at 32 kHz (Fig. 6a). At 50 kHz, the main frequency peak ranges from 10.69 dB to 29.50 dB, the second harmonic peak from 4.53 dB to 18.44 dB, and the peak at LPR from 4.14 dB to 19.72 dB (Fig. 6b). This indicates a comparable dynamic range for both LPRs.

The steady parallel growth of the fundamental frequency peak and additional peaks indicates a strong correlation between the fundamental frequency signal and the additional peaks. As the fundamental frequency increases, interference increases proportionally with the signal, maintaining a steady relationship throughout the range. At 8kHz LMR /32 kHz LPR, the SNR at the LPR is typically higher than at f_0_. For the 50 kHz LPR stimulation, the SNR at f_0_ was consistently higher than at both additional peaks. When normalized to the $f_{0}$-SNR, the peak at the 50 kHz LPR remained between 0.35 and 0.80 of the main peaks across all power levels, indicating that the growth of the artifacts was proportional to the signal itself for 50 kHz LPR. This proportional increase is also reflected in the stable differences between $f_{0}$-SNR and the second harmonic or LPR-SNR values.

#### Optical to acoustic level mapping in vitro

**Fig. 7:**
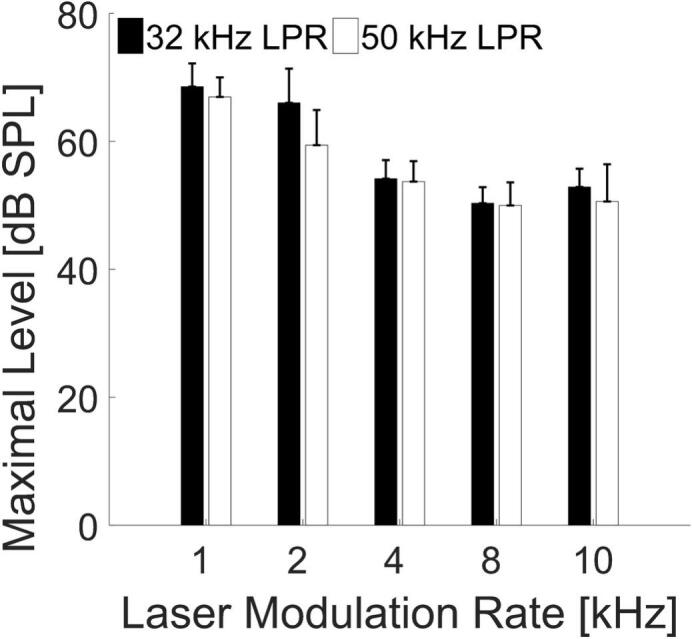
Level Mapping in vitro Graph presenting the maximal sound pressure levels (SPL) equivalent measured during optical (laser) and acoustic stimulation at different laser modulation rates. The black bars represent the 32 kHz laser pulse rate (LPR) and the white bars the 50 kHz LPR.

Overall, the maximal SPL compared to acoustic stimulation occurs at the lowest modulation rate of 1 kHz, reaching 68.56 dB SPL (SD = 3.63 dB SPL) for 32 kHz LPR and 66.02 dB SPL (SD = 5.34 dB SPL) for 50 kHz. The minimal SPL is observed at 8 kHz, with 50.34 dB SPL (SD = 2.5 dB SPL) at 32 kHz LPR and 49.90 dB SPL (SD 3.62 dB SPL) at 50 kHz LPR. Across all modulation rates, the SPL decreases with the modulation rate from 1 kHz to 8 kHz, followed by a slight increase at 10 kHz (Fig. 7).

### *In vivo* recordings at the ICC level

#### Growth functions driven spike rate vs. peak power

We calculated the equivalent sound pressure for the maximal displacement at f_0_ for each animal.

$$x\;dB\;SPL=20\cdot {log}_{10}\left(\frac{\hat{y}}{{\hat{y}}_{60\;dB}}\right)+60\;dB\;SPL$$

**Fig. 8:**
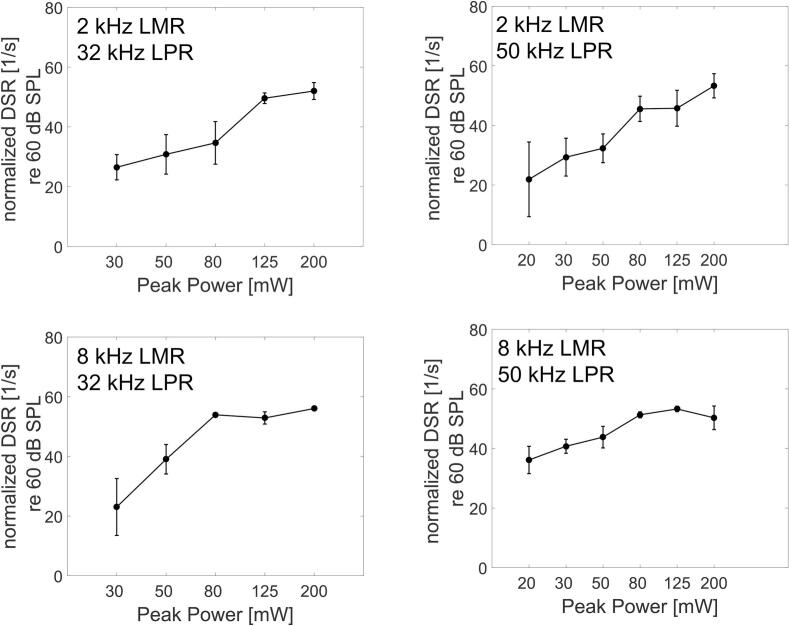
Growth functions in vivo Normalized growth function demonstrating the steady growth of driven spike rate (DSR) for increasing laser powers at different laser modulation rate/laser pulse rate combinations.

The modulation of the peak amplitude at f_0_ is herein demonstrated at 2 kHz LMR and 8 kHz combined with 32 and 50 kHz LPR. The equivalent sound pressure level for the amplitude of f_0_ of the tested frequencies was calculated as described relative to 60 dB SPL (Fig. 8).

With the laser peak power, the sound level equivalent increases with a steep slope up to 80 mW, followed by a shallower slope after 80 mW peak power. In a few animals, better activation at 80 mW could be observed.

#### Optical to acoustic level mapping in vivo

**Fig. 9:**
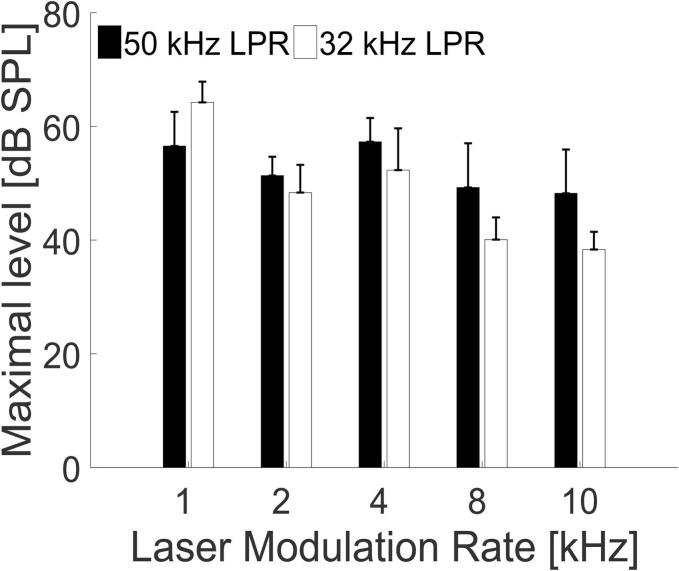
Level Mapping in vivo Optically induced driven spike rates compared to acoustic-driven spike rates, expressed as equivalent dB SPL, for different laser modulation rate/laser pulse rate combinations.

Overall, the maximal acoustic equivalent SPL occurs at 1 kHz LMR and 32 kHz LPR (64.21 dB SPL), followed by 56.53 dB SPL at 50 kHz LPR. The closest minimal SPL is observed at 10 kHz, with 38.33 dB SPL at 32 kHz LPR and 48.22 dB SPL at 50 kHz LPR. Across all modulation rates, the SPL decreases with the modulation rate varied from 1 kHz to 10 kHz, for both LPR (Fig. 9).

## Discussion

This study confirms that optoacoustic stimulation of the (TM) can elicit reliable auditory responses, both peripherally and centrally, across a range of laser powers. By combining *in vitro* LDV measurements on guinea pig temporal bones with *in vivo* recordings in the ICC midbrain, we demonstrate a consistent and scalable transmission of laser-induced mechanical energy into the auditory system.

Previous work has already proven the potential of frequency-specific activation of the auditory pathway using modulated green laser stimulation of the TM [13]. Based on this knowledge, our data provides important new insights into how these frequency-specific responses scale with optical power. We observed a linear growth function between 30 mW and 200 mW for multiple modulation frequencies, both in terms of membrane displacement (*in vitro*) and neural response strength (*in vivo*). This linearity confirms the system’s potential for controlled, dynamic stimulation, a key prerequisite for the development of a laser-based hearing aid.

Importantly, this linear relationship remains for all tested frequencies, 1, 2, 4, 8, and 10 kHz, indicating that frequency-specific stimulation remains robust over a wide range of laser intensities. The growth functions for each frequency showed similar slopes, suggesting a largely uniform sensitivity of the TM and auditory system to the optoacoustic stimulus across the auditory spectrum. No indications of saturation or non-linear effects were observed within the tested power range up to 200 mW, supporting the feasibility of delivering frequency-multiplexed stimulation with predictable output characteristics.

A parallel increase in additional spectral components, particularly at the second harmonic and at the laser pulse repetition rate, was also observed. These components increased proportionally with the peak at the fundamental frequency, indicating that they are inherent to the optoacoustic mechanism rather than due to system distortion. While this proportional behavior does not compromise linearity per se, it may impose constraints on SNR, especially in low-intensity applications or high-frequency stimulation. The pulse repetition rates employed here (32 kHz and 50 kHz) are far beyond the upper limit of human auditory perception (<20 kHz). Consequently, the LPR acts as an inaudible optical carrier and is not expected to produce any direct perceptual sensation. Only the frequency content imposed on the optical envelope by the intensity-modulation scheme falls within the audible range. This distinction is essential for auditory-prosthesis design, as it implies that the carrier rate can be chosen for optimal optoacoustic efficiency and thermal safety without affecting the perceived pitch. It also ensures that stimulation strategies can remain compliant with human auditory constraints while exploiting high-frequency carrier pulses for stable and safe optoacoustic generation. Applying a different modulation strategy, like an adapted pulse density modulation, could further reduce the effect of the carrier frequency.

To understand why linear scaling is preserved across a wide range of stimulation intensities, we examined the impulse-level mechanical response of the TM and related the extracted parameters to a reduced thermoelastic framework. Our previous study demonstrated frequency-specific modulation of the auditory pathway using amplitude-modulated laser stimulation [13], but the physical mechanism enabling this behavior is not fully understood. Here, we address this mechanism by quantifying both the static sensitivity and the intrinsic temporal dynamics of the membrane from single-pulse LDV recordings and linking these parameters directly to optical energy deposition.

Within the thermoelastic confinement regime, absorbed pulse energy produces transient thermal expansion that generates mechanical displacement while leaving the tissue’s structural properties unchanged. In line with this prediction, the compliance values derived from single-pulse peak amplitudes remained largely stable across all tested intensities, indicating that increased laser drive scales displacement, but does not modify the effective mechanical stiffness of the membrane. Similarly, the temporal decomposition of the displacement waveform revealed intensity-independent relaxation and oscillation time constants. These invariant parameters show that, within the studied regime, the TM acts as a linear mechanical system.

This provides a physical explanation for the uniform scaling of frequency-modulated responses with laser power: the modulated output effectively corresponds to a scaled version of a characteristic mechanical response of the TM. Thus, the frequency-specific responses observed here and previously can be interpreted as the convolution of the optical envelope with a power-invariant mechanical impulse response of the tympanic membrane, as suggested in [13]. Establishing this link between optical input, membrane mechanics, and auditory activation provides a physically grounded framework for designing multi-frequency, power-scalable optoacoustic stimulation strategies.

Optoacoustic stimulation of the peripheral hearing system offers several advantages over conventional hearing aids, which rely on amplified airborne sound and are therefore limited by middle-ear pathologies, acoustic feedback, and cochlear nonlinearities. As the laser induces vibration directly at the TM via thermoelastic expansion, it avoids feedback and occlusion, preserves natural pinna cues, and delivers mechanical energy efficiently into the ossicular chain. The linear growth functions observed across frequencies and intensities further distinguish this approach from classical amplification systems that depend on multiband compression. Optoacoustic stimulation remained linear in both mechanical and neural responses, and we expect cleaner and more predictable stimulus coding with fewer distortions.

These benefits, however, do not overcome all constraints of sensorineural hearing loss. Pathologies located within the cochlea, such as dead regions, severe loss of inner or outer hair cells, and degeneration of spiral ganglion neurons, cannot be compensated for by increasing mechanical drive at the TM. Likewise, optoacoustic stimulation cannot restore normal cochlear frequency selectivity or mitigate intrinsic cochlear nonlinearities when outer hair cell function is compromised.

Regarding potential human application, laser safety limits must be carefully considered. Previous work reported no adverse effects up to 89 mW [15], whereas the power levels used in the present study exceeded common safety thresholds and were therefore restricted to *ex vivo* and *in vivo* animal experiments. Translating this approach to human use will require either reduced pulse energies, shorter pulses, improved optical focusing, or more efficient absorbers to remain within permissible exposure levels. First investigations using an absorber patch applied directly to the TM demonstrated that such an interface can enhance local laser absorption and maintain the linearity of the amplitude modulation while simultaneously protecting the irradiated tissue [16]. These results suggest that optimized absorber designs may play a key role in enabling safe and effective human-oriented optoacoustic stimulation.

Overall, optoacoustic stimulation represents a promising alternative mechanism where traditional acoustic hearing aids are limited by middle-ear mechanics, altered ear-canal acoustics, or feedback susceptibility; however, its applicability remains constrained by cochlear pathologies, optical coupling efficiency at the TM, and safety limits associated with laser-based stimulation.

In summary, our findings support the feasibility of multi-frequency, power-scalable stimulation paradigms and represent an important step toward the development of future auditory prostheses.

Ongoing work focuses on miniaturizing the laser and processing units and on further optimizing modulation strategies for practical implementation.

## Conclusion

This study focuses specifically on the modulation of laser intensity. We demonstrate that TM vibrations increase linearly with optical power and elicit correspondingly stronger neuronal responses in the inferior colliculus. By relating the experimentally determined mechanical response of the tympanic membrane to basic thermoelastic principles, we provide a physically motivated interpretation of this linear relationship: absorbed optical energy induces transient local temperature changes that result in measurable membrane displacements, which are directly reflected in the observed neural activation.

This combination of experimental measurements and physically guided analysis represents an important step toward the development of light-based auditory stimulation approaches. Looking ahead, potential fluctuations in stimulation intensity could be mitigated through optical modifications of the TM, such as the application of a protective absorber patch, which may both stabilize the mechanical response and protect the tissue from excessive light exposure. The present analysis further supports the definition of safe and effective stimulation regimes and may guide future designs of laser-based auditory prostheses for human application.

## Grants and funding

This research has been funded by the European Research Council under the European Union’s Seventh Framework Program (FP/2007-2013)/ERC Grant, LaserHearingAids: 311469.

## CRediT authorship contribution statement

**Patricia Dries:** Writing – original draft, Software, Methodology, Investigation, Data curation, Conceptualization. **Hubert H. Lim:** Writing – review & editing, Validation, Software, Methodology. **Marius Hinsberger:** Writing – review & editing, Investigation. **Katharina Sorg:** Writing – review & editing, Investigation. **Lukas Pillong:** Writing – review & editing, Investigation. **Achim Langenbucher:** Writing – review & editing, Supervision, Methodology. **Bernhard Schick:** Writing – review & editing, Supervision, Resources. **Gentiana I. Wenzel:** Writing – review & editing, Supervision, Resources, Methodology, Investigation, Conceptualization.

## Declaration of competing interest

The authors declare that they have no known competing financial interests or personal relationships that could have appeared to influence the work reported in this paper.
